# Home-based Testing for SARS-CoV-2: Leveraging Prehospital Resources for Vulnerable Populations

**DOI:** 10.5811/westjem.2020.5.47769

**Published:** 2020-06-15

**Authors:** Scott A. Goldberg, Robert A. Bonacci, Lucas C. Carlson, Charles T. Pu, Christine S. Ritchie

**Affiliations:** * Brigham and Women’s Hospital, Department of Emergency Medicine, Boston, Massachusetts; † Population Health Management, Partners HealthCare, Boston, Massachusetts; ‡ Massachusetts General Hospital, Department of Medicine, Boston, Massachusetts; § Massachusetts General Hospital, Department of Medicine, Division of Palliative Care and Geriatric Medicine and the Mongan Institute, Boston, Massachusetts

## Abstract

**Introduction:**

Expanded testing for SARS-CoV-2 is critical to characterizing the extent of community spread of COVID-19 and to identifying infectious cohorts. Unfortunately, current facility-based testing compounds shortcomings in testing availability, neglecting those who are frail or physically unable to travel to a testing facility.

**Methods:**

We developed an emergency medical service (EMS)-based home testing and evaluation program, leveraging existing community EMS resources. This program has kept vulnerable populations out of the emergency department, reduced cost, and improved access to care.

**Results:**

Our EMS-based testing program can test approximately 15 homebound patients per day. Through April 2020 our program had performed 477 home-based tests. Additionally, we have recently undertaken several mass testing operations, testing up to 900 patients per testing site.

**Conclusion:**

Facility-based SARS-CoV-2 testing requires that a patient physically present to a facility for a nasopharyngeal swap to be collected. Unfortunately, access may be limited for patients that are homebound, chronically ill, or without a means of private transportation. By leveraging existing EMS infrastructure in new ways, our community has been able to keep almost 500 vulnerable patients in their home. Using EMS, we can strengthen the healthcare system’s response to the evolving COVID-19 pandemic and support at-risk populations, including those that are underserved, homebound, and frail.

## INTRODUCTION

COVID-19, the disease caused by the novel coronavirus SARS-CoV-2, has rapidly developed into a global pandemic affecting millions of people across the world. As the pandemic spreads throughout the United States, healthcare systems simultaneously face increasing demand for services and constraints on resources available for laboratory testing, inpatient care, and infection control. Expanded access to testing in particular is critical to characterizing the extent of community spread and recognizing infectious cohorts.^1^ Delays in testing can lead to devastating consequences, and insufficient testing was identified as a critical shortcoming in the early response to SARS-CoV-2 in China and the US compared to countries with more aggressive testing programs.^2^–^5^ In February 2020 delayed recognition was at least partially responsible for the spread of the virus to 81 residents at a nursing facility in Washington State, resulting in 23 deaths.^6^

Current facility-based practices of testing for SARS-CoV-2 further compound present shortcomings in testing availability. In the US 9% of households do not have access to a private vehicle and more than 15% of older adults are characterized as frail and may be physically unable to travel to a facility for testing without assistance.^7^ Almost 6% of the Medicare population is mostly or completely homebound.^8^ These persons often rely on the assistance of others, who may either serve as a vector for COVID-19 or may inadvertently become infected by the homebound populations they serve. Inability to test homebound adults may not only have a role increasing spread to household members but also to personal care assistants taking care of other vulnerable populations at the same time. Furthermore, individuals facing related barriers to facility-based testing are often those most vulnerable to COVID-19 due to existing medical co-morbidities and socioeconomic risk. The evolving pandemic will likely further amplify existing barriers to healthcare, especially among people of advanced age and populations who are poor, homeless, or living with chronic illness, populations known to have a higher mortality rate from COVID-19.^6^ Further, these communities are more likely to be disconnected from medical care, lack safe places for shelter, and face financial strain if they become ill.

## METHODS

Recognizing these inefficiencies and shortcomings, we designed an emergency medical services (EMS)-based SARS-CoV-2 home testing and evaluation program in partnership with local ambulance agencies. The goal of this program was to efficiently expand access to SARS-CoV-2 testing, leveraging a resource already available in most communities in the US. The use of EMS providers to respond to novel healthcare needs, termed mobile integrated healthcare (MIH), has been widely described in a variety of contexts. EMS providers have local ties and cultural competency similar to community health workers coupled with a high level of medical knowledge and procedural skills. Further, EMS providers are well versed in the evaluation and management of this complicated patient population, able to leverage their unique insights to identify subtle changes in patients’ health status, making them well-suited and uniquely prepared to serve the communities that stand to be most dramatically impacted by the COVID-19 pandemic.

In the early days of the COVID-19 pandemic, our hospital system worked closely with a partner EMS agency to develop a mobile testing program. As part of emergency provisions pertaining to the COVID-19 response, the Massachusetts Department of Public Health added the skill of nasopharyngeal swab collection to our 2020 Statewide Treatment Protocols as acceptable practice for providers at the emergency medical technician (EMT) or paramedic level. Initially, six paramedics were trained in the skill of nasopharyngeal swab collection for polymerase chain reaction analysis, specimen handling, and safe use of specialized personal protective equipment (PPE). We were able to quickly scale this workforce to 17 EMTs and paramedics over the first week of the program. Our standard training was of two hours duration, after which all providers were able to demonstrate proficiency not only in the skill of nasopharyngeal swab collection, but in the process of responding to a dispatch for SARS-CoV-2 testing as well.

## RESULTS

In our program, after a physician or advanced practice provider deems that a patient requires testing based on symptoms and epidemiological or occupational risk factors, an EMS provider is deployed to the patient’s home to collect a nasopharyngeal swab and then transports the specimen to the appropriate laboratory testing facility. EMS providers work in a team of no less than two, allowing for a monitor to carefully observe specimen collection for any PPE breeches, and to collect handoff of the specimen into a sterile receptacle after collection. The technician collecting the sample wears recommended PPE including an N95 mask, eye protection, an impermeable gown, and gloves. Gloves are changed after each patient encounter and gowns are changed any time the technician interacts with a new environment, in line with guidelines for facility-based testing. A mobile team of two providers can test approximately 15 homebound patients per day, depending on geographic distances. A larger team can test several hundred in highly orchestrated mass-testing operations. We currently operate up to two teams of two paramedics per day testing up to 20 patients, depending on the geographic distance covered. If the testing radius is small only two paramedics are needed.

Unfortunately, laboratory testing in the home introduces operational inefficiencies depending on patient geography. While the home visit to obtain the nasopharyngeal swab is quick, the team spends significant time traveling point to point. Most of our patient reside within a small geographic area, taking our paramedics an estimated 20 minutes to respond to an individual address. However, some patients reside up to an hour from our main hospital campus. These distances are mitigated by cohorting patients with similar geographic distributions, with one EMS testing unit responding to a geographic cluster of patients.

Like many healthcare systems with limited access to and supply of testing materials for COVID-19, our system’s testing criteria are rapidly evolving. To date, the majority of patients tested through our program have been symptomatic. However in recent weeks, as access to testing capacity has increased, we have begun testing certain asymptomatic individuals as well. One such example includes COVID-19 positive patients needing clearance to return to hemodialysis. As of April 30, our program has performed 477 home-based tests.

In addition to our home-based mobile testing operation, we have recently undertaken several mass-testing operations, using a larger team of six providers to test 900 patients at a single site over eight hours. These campaigns include sites such as senior living communities and skilled nursing facilities. Our Department of Public Health had leveraged our program to test over 11,667 patients as of April 30 at 941 facilities across the Commonwealth of Massachusetts.^9^

## DISCUSSION

Facility-based SARS-CoV-2 testing, as employed within our system, requires a patient to physically present to a facility for a nasopharyngeal swab to be collected. Some of these facilities are traditional healthcare settings such as hospitals, while others such as “drive-through” testing sites may be more accessible. However, in almost all cases access is limited, particularly for patients who are homebound, chronically ill, or without a means of private transportation. Use of rideshare or public transportation is not possible due to the risk of spreading infection, and public transportation is similarly not an option. Lacking other viable options, these high-risk patients were transported to the facility by ambulance – a solution that was inefficient and costly, took ambulances offline and unable to respond to our community, and put additional healthcare workers at risk of exposure.

Leveraging community resources and employing an EMS provider model improves healthcare resource utilization by 1) ensuring that patients who do not need acute care are diverted away from crowded emergency departments and ambulatory clinics; 2) maximizing ambulance operational time; 3) reducing cost; and 4) accessing medically challenging and traditionally underserved patient populations. Future considerations for employing MIH programs for patients with COVID-19 include incorporating remote monitoring capabilities and outpatient home visits, as well as expanding operations of hospital-at-home programs offering inpatient level care at home. These efforts are designed to augment the health system’s capacity to deliver acute care services and meet the escalating demands of the COVID-19 pandemic. Furthermore, we believe these efforts will be sustained and extend any organization’s prior value-based care journey and which the COVID-19 pandemic has only accelerated.

## LIMITATIONS

Our special pathogens home-testing program is subject to a few limitations. First, the program is currently limited to testing only, without incorporating home monitoring or treatment for COVID-19. Second, at-home testing introduces unavoidable inefficiencies. Mass testing is more efficient but can be challenging to coordinate, especially while complying with physical distancing restrictions. This service is also primarily hospital funded. While the Centers for Medicare and Medicaid Services has suggested that there may be reimbursement for collection of SARS-CoV-2 tests for patients unable to travel, how or whether this will be implemented remains unclear. Finally, we do not yet have results to report for our patients and the prevalence of COVID-19 in our community may not be generalizable. As of April 30, our community had a positivity rate of 19% of patients who were tested.^9^

## CONCLUSION

While this EMS-based home testing program created for COVID-19 is one of the first of its kind in the US, it will certainly not be the last. Healthcare organizations across the US can prepare for this rapidly expanding pandemic through the use of EMS and MIH to support diagnosis as well as evaluation, monitoring, and treatment of COVID-19. By leveraging the existing EMS infrastructure in new ways, our community has been able to keep almost 500 vulnerable patients in their homes. We have further been able to support our public health infrastructure by testing thousands of residents of vulnerable facilities including nursing homes, assisted living facilities, and other state-run facilities. Using EMS, we can strengthen the healthcare system’s response to the evolving COVID-19 pandemic and support at-risk populations, such as those that are underserved, homebound, or frail. These resources are already available in our communities and in the face of this pandemic, there is no time to wait.
